# Pulmonary Flora‐Derived Lipopolysaccharide Mediates Lung‐Brain Axis through Activating Microglia Involved in Polystyrene Microplastic‐Induced Cognitive Dysfunction

**DOI:** 10.1002/advs.202404966

**Published:** 2024-11-05

**Authors:** Huiwen Kang, Danyang Huang, Wei Zhang, JingYu Wang, Ziyan Liu, Ziyan Wang, Guangyu Jiang, Ai Gao

**Affiliations:** ^1^ Department of Occupational Health and Environmental Health, School of Public Health Capital Medical University Beijing 100069 China; ^2^ Beijing Key Laboratory of Environmental Toxicology Capital Medical University Beijing 100069 China

**Keywords:** cognitive, LPS, lung‐brain axis, microplastics, pulmonary flora

## Abstract

Microplastics (MPs) have been detected in the atmospheric and the human respiratory system, indicating that the respiratory tract is a significant exposure route for MPs. However, the effect of inhaled MPs on cognitive function has not been adequately studied. Here, a C57BL/6 J mouse model of inhalation exposure to polystyrene MPs (PS‐MPs, 5 µm, 60 d) is established by intratracheal instillation. Interestingly, in vivo fluorescence imaging and transmission electron microscopy reveal that PS‐MPs do not accumulate in the brain. However, behavioral experiments shows that cognitive function of mice is impaired, accompanied by histopathological damage of lung and brain tissue. Transcriptomic studies in hippocampal and lung tissue have demonstrated key neuroplasticity factors as well as cognitive deficits linked to lung injury, respectively. Mechanistically, the lung‐brain axis plays a central role in PS‐MPs‐induced neurological damage, as demonstrated by pulmonary flora transplantation, lipopolysaccharide (LPS) intervention, and cell co‐culture experiments. Together, inhalation of PS‐MPs reduces cognitive function by altering the composition of pulmonary flora to produce more LPS and promoting M1 polarization of microglia, which provides new insights into the mechanism of nerve damage caused by inhaled MPs and also sheds new light on the prevention of neurotoxicity of environmental pollutants.

## Graphical abstract

Microplastics (MPs) have been detected in the human respiratory system, indicating that the respiratory tract is a significant exposure route for MPs. Pulmonary flora‐derived lipopolysaccharide mediates lung‐brain axis through activating microglia, which is one of the mechanisms behind cognitive dysfunction induced by inhalation of MPs. Inhibiting LPS offer a preventive strategy against nerve injury caused by inhalation of MPs.

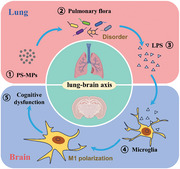

## 1 Introduction

Microplastics (MPs) are ubiquitous across various environmental media due to the extensive global use and slow degradation of plastics. With evidence emerging of MPs being detected in human lung tissue,^[^ ^1^, ^2^ ^]^ nasal lavage fluid, and sputum,^[^ ^3^, ^4^ ^]^ as one of the important pathways of MPs intake, the harm of respiratory intake to the body has gradually attracted wide attention. Airborne MPs can originate from sources such as synthetic textile fibers, sewage, landfills, and tire wear.^[^ ^5^ ^]^ Despite the serious pollution of MPs in the air, the concentration of airborne MPs has not been accurately quantified. A study have found that the concentration of MPs in the air in northern Chinese cities was 358 ± 132 MPs m^−3^ higher than 230 ± 94 MPs m^−3^ in southeastern cities.^[^ ^6^ ^]^ A review estimated that the concentration of MPs ranges from 1 to 1000 MPs m^−3^ in outdoor air and from 1 to 1583 ± 1181 MPs m^−3^ in indoor air.^[^ ^7^ ^]^ In addition, it has the characteristics of small particle size and strong adsorption, and long‐term inhalation of air containing MPs may cause harm to a variety of organs and systems of the body.

The nervous system is one of the most complex and sensitive systems in the human body, playing a crucial role in maintaining normal bodily functions. Exposure to air pollutants has been reported to affect nervous system function, including inducing depression,^[^ ^8^ ^]^ mood disorders,^[^ ^9^ ^]^ and cognitive impairment.^[^ ^10^, ^11^ ^]^ The potential damage to the nervous system caused by emerging environmental pollutants has attracted widespread concern. Currently, MPs have not been detected in the human brain, but animal experimental studies have found that the ingestion of MPs via the digestive tract may cause nerve damage, disrupt the blood‐brain barrier (BBB) of mice,^[^ ^12^ ^]^ induce the activation of brain microglia and neuronal damage,^[^ ^13^ ^]^ and lead to dose‐dependent cognitive decline.^[^ ^14^, ^15^, ^16^ ^]^ Importantly, few studies have investigated nerve damage caused by inhalation of MPs in mammals, and the specific mechanisms underlying this damage remain unknown. Identifying the key processes involved could provide valuable insights into the prevention and treatment of neurotoxicity caused by inhalation of MPs. Previous epidemiological and animal studies have identified the lungs as a primary target of air pollution's harmful effects.^[^ ^17^ ^]^ Numerous studies support a close relationship between lung injury and nervous system function. For example, individuals with restrictive or obstructive lung impairment have a higher risk of dementia.^[^ ^18^ ^]^ Smoking and pulmonary infections can increase the risk of multiple sclerosis.^[^ ^19^ ^]^ Fine particulate matter (PM_2.5_) has been shown to cause cognitive decline in mice.^[^ ^20^ ^]^ It is speculated that MPs inhaled through the respiratory tract may affect nervous system function by causing lung damage.

The concept of the lung‐brain axis was proposed by Alexander Flugel et al. in 2022, with pulmonary flora playing a significant role. In a rat model of experimental pulmonary autoimmune encephalomyelitis, they found that pulmonary flora could exert an immunomodulatory effect by producing more lipopolysaccharide (LPS) and influencing microglia in the brain.^[^ ^21^ ^]^ It has also been suggested that the vagus nerve may be a key mediator of the lung‐brain axis. Mesenchymal stromal cells induce the release of serotonin (5‐HT) in the brain's dorsal nucleus, alleviating depression and anxiety‐like behaviors by activating pulmonary sensory neurons that innervate the vagus nerve, which then project to the nucleus of the solitary tract.^[^ ^22^ ^]^ The human microbiota consists of all microorganisms colonizing the human body, primarily distributed in the skin, intestines, lungs, and other areas. In recent years, the influence of gut microbiota on various diseases has been well established, but studies on pulmonary flora remain limited. Accumulating evidence suggests that pulmonary flora plays a crucial role in promoting lung health. Disorder of pulmonary flora is a core contributor to asthma, allergic diseases,^[^ ^23^ ^]^ and chronic obstructive pulmonary disease.^[^ ^24^ ^]^ In addition, one study found that 23.7% of lung lesions were associated with changes in pulmonary flora.^[^ ^25^ ^]^ Recent studies have shown that respiratory exposure to MPs can cause an imbalance in pulmonary flora.^[^ ^26^, ^27^ ^]^ Therefore, we hypothesize that the lung‐brain axis, mediated by an imbalance in pulmonary flora, could be a potential mechanism of nerve injury induced by inhaled MPs.

In this study, a mouse model of PS‐MPs inhalation exposure was established via tracheal instillation. It was found that PS‐MPs (5 µm) did not accumulate in the brain, but cognitive function of the mice was decreased. Through RNA sequencing (RNA‐seq) and 16 s ribosomal RNA (rRNA) analysis revealed that inhalation of PS‐MPs induced lung injury, and pulmonary dysbiosis may be involved in the process of cognitive decline. Focusing on the lung‐brain axis, it is speculated that increased LPS production caused by pulmonary flora disorder and M1 polarization of microglia may play a key role in PS‐MPs‐induced cognitive impairment. Pulmonary flora transplantation (PFT), LPS intervention, and cell co‐culture experiments were conducted to validate the results. These findings aim to identify effective intervention targets for cognitive impairment‐related diseases caused by MPs respiratory exposure and to provide a theoretical basis for the risk assessment and early warning of neurotoxicity in populations exposed to MPs.

## 2 Results

### 2.1 Characterization of PS‐MPs

**Figure 1:**
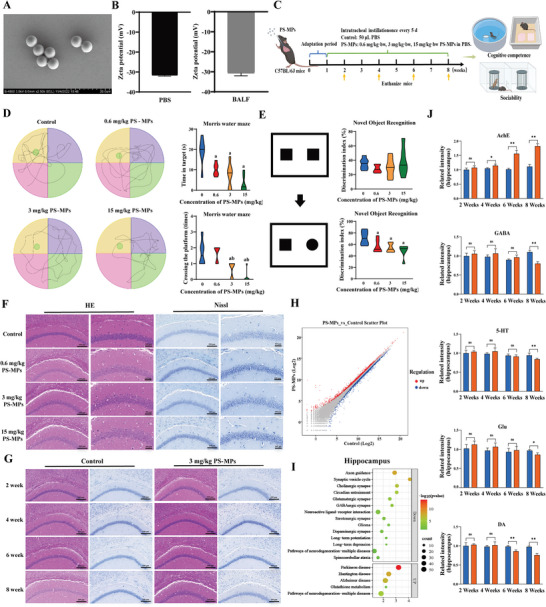
Effect of PS‐MPs exposure on cognitive function in mice. A) The morphology of 5 µm PS‐MPs. B) Zeta potentials of PS‐MPs in PBS or BALF. C) Schematic diagram of animal experiment. D) The spatial localization learning and memory ability of mice was tested by morris water maze test (*n* = 6). E) The non‐spatial learning and memory ability of mice was tested by novel object recognition test (*n* = 6). F) Effects of different concentrations of PS‐MPs on the hippocampal CA1 region of mice. G) The changes of hippocampal CA1 region in mice after 2 weeks (w), 4 w, 6 w, and 8 w of PS‐MPs (3 mg kg^−1^) exposure. H) The distribution of differential mRNA of hippocampal (*n* = 4). I) Differentially expressed signaling pathways obtained by KEGG enrichment analysis. J) Expression of important neuroplasticity‐related molecules in differentially expressed signaling pathways in the hippocampus (*n* = 5). ^ns^ *p* > 0.05, *p < 0.05, **p < 0.01. MPs: microplastics; BALF: bronchoalveolar lavage fluid; AChE: acetylcholinesterase; GABA: γ‐aminobutyric acid; 5‐HT: Serotonin; Glu: glutamate; DA: dopamine; KEGG: kyoto encyclopedia of genes and genomes.

The 5 µm PS‐MPs particles exhibited a regular, uniform spherical shape under SEM examination (**Figure** **1**A). The zeta potential and hydrodynamic size of PS‐MPs in PBS were ‐31.63±0.45 mV (Figure 1B) and 5125.33±242.17 nm (Figure S1A,B, Supporting Information), respectively. In addition, the zeta potential of PS‐MPs in bronchoalveolar lavage fluid (BALF) was ‐30.63±1.38 mV (Figure 1B). These results indicate that the PS‐MPs used in this study exhibit good stability and dispersion both before intratracheal instillation and within the lungs of mice.

### 2.2 Inhalation of PS‐MPs Can Reduce Cognitive Function in Mice

Mice were intratracheally instilled with three concentrations of PS‐MPs (0.6 mg kg^−1^, 3 mg kg^−1^, and 15 mg kg^−1^) for 60 days (d) (Figure 1C). No significant differences in body weight were found between groups at each time point (2 weeks (w), 4 w, 6 w, and 8 w) (Figure S1C, Supporting Information). Behavioral testing was performed at 8 w. In the Morris water maze (MWM) test, there was no difference in swimming speed among the three PS‐MPs‐exposed groups compared to the control group during the 4 d training period, and the escape latency in each group showed a gradually decreasing trend. On day 4, the escape latency and total swimming distance were greater in the PS‐MPs‐exposed mice than in the control mice (Figure S1D, Supporting Information). On the fifth day of testing, the time spent in the target quadrant was reduced in all three PS‐MPs‐exposed groups compared to the control mice. The 0.6 mg kg^−1^ PS‐MPs group did not significantly affect the time spent in the target quadrant, while the 3 mg kg^−1^ and 15 mg kg^−1^ PS‐MPs groups showed a reduced time in the target quadrant (Figure 1D). In the Novel object recognition (NOR) test, there was no significant difference in the recognition index of mice at the beginning of the experiment. After changing the new object, the recognition index of mice in the three PS‐MPs exposure groups decreased (Figure 1E). In the three‐chamber sociability test, there were no significant differences in the duration and number of contacts between all mice and E and S1 and between S1 and S2 (Figure S1E, Supporting Information). In conclusion, inhalation of PS‐MPs can reduce the cognitive function of mice, but has no significant effect on social ability.

The hippocampal formation is fundamental to memory formation and spatial cognition.^[^ ^28^, ^29^ ^]^ Next, we examined the histopathological changes in the hippocampus of mice, including Hematoxylin‐Eosin (H&E staining) and Nissl staining (Figure 1F,G). After 8 w of PS‐MPs exposure, the cells in the hippocampal CA1 region of the three PS‐MPs groups were loosely arranged, some nuclei were pyknotic, neuron loss occurred, and the damage was more obvious with the increase of PS‐MPs concentration. To observe the hippocampal injury of mice with different exposure times, the pathological changes of the hippocampal CA1 region at different time points (3 mg kg^−1^) were further detected. Compared with the control group, the cells showed loose arrangement, but there was no significant neuronal loss at 2 w. The looseness of the cells was relieved, but there was a marked pyknosis of the cells at 4 w. Some nuclei were pyknotic and some neurons were lost at 6 w. The results were the same as previously described, with aggravated damage at 8 w. Therefore, inhalation of PS‐MPs can damage the hippocampus, and the damage is aggravated with the increase in exposure time.

To discover the possible signaling pathways affected by PS‐MPs exposure, we performed an RNA‐seq analysis of hippocampal tissues. Kyoto Encyclopedia of Genes and Genomes (KEGG) enrichment analysis of differential genes (Figure 1H) showed that 14 down‐regulated pathways and 5 up‐regulated pathways were closely related to cognition (Figure 1I). Neuroplasticity is an adaptive change in the structure and function of the nervous system.^[^ ^30^ ^]^ Neurotransmitters and related enzymes can affect cognitive function by affecting the neuroplasticity of the hippocampus. The analysis found that acetylcholinesterase (AChE), as well as four neurotransmitters (GABA, 5‐HT, Glu, and DA), is the key factor of nerve injury induced by inhalation of PS‐MPs. The expressions of the five factors in the hippocampus and plasma at different time points were detected, and the expressions of the five factors in hippocampus and plasma were changed in the 8 w (Figure 1J; Figure S1F, Supporting Information), which was consistent with the behavioral results.

### 2.3 The Lung‐Brain Axis is an Important Cause of Neurotoxicity Induced by PS‐MPs

**Figure 2:**
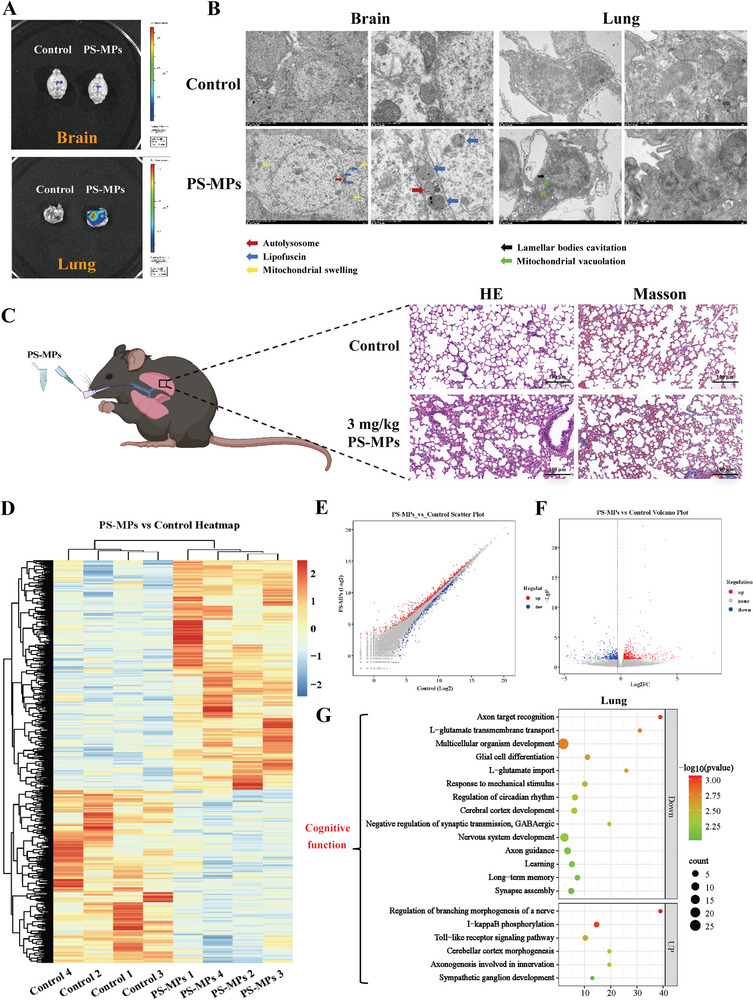
Inhalation of PS‐MPs may cause cognitive impairment through lung injury. A) Distribution of inhaled PS‐MPs in lung and brain. B) Ultrastructural destruction of brain and lung tissues. C) PS‐MPs caused lung tissue damage. D–F) Hierarchical clustering and distribution of differential mRNAs in lung tissue after PS‐MPs exposure (*n* = 4). G) Differentially expressed signaling pathways obtained by KEGG enrichment analysis.

The nerve injury caused by inhalation of PS‐MPs may not be a direct effect of PS‐MPs, but an indirect effect mediated by other substances or pathways. The distribution of PS‐MPs in various organs was observed 8 w after tracheal instillation of 5 µm PS‐MPs containing fluorescence (Figure S2A, Supporting Information). We did not observe obvious PS‐MPs in the brain tissue, but found the presence of PS‐MPs in the lung (**Figure** **2**A; Figure S2B, Supporting Information). By ultrastructural examination, we found mitochondrial swelling, autolysosomes, and lipofuscin deposition in the hippocampal CA1 region of mice after PS‐MPs exposure. Vacuolization of mitochondria and lamellar bodies was observed in lung tissue. Although previous studies have reported that 50 nm MPs can enter the intracellular of lung tissue.^[^ ^31^ ^]^ However, because of the large size of 5 µm MPs, it is difficult to enter lung tissue cells, so PS‐MPs particles were not found in the cells (Figure 2B).

Due to the exposure through the respiratory tract, the lung is one of the important target organs of PS‐MPs. Studies have found that the pathophysiology of lung‐brain interaction is complex, involving immune responses, inflammation, and so on.^[^ ^32^ ^]^ Histopathological examination showed that inhalation of PS‐MPs caused increased inflammatory cell infiltration, interstitial and alveolar edema, rupture of lung septum, and increased collagen deposition in lung tissues of mice (Figure 2C). Differentially expressed genes after PS‐MPs exposure were detected by RNA‐seq (Figure 2D–F), and KEGG enrichment analysis revealed 14 down‐regulated and 6 up‐regulated pathways related to cognitive function, which also contained the previously mentioned neuroplasticity related factors (Figure 2G). The lung‐brain axis is involved in the process of nerve injury induced by PS‐MPs.

**Figure 3:**
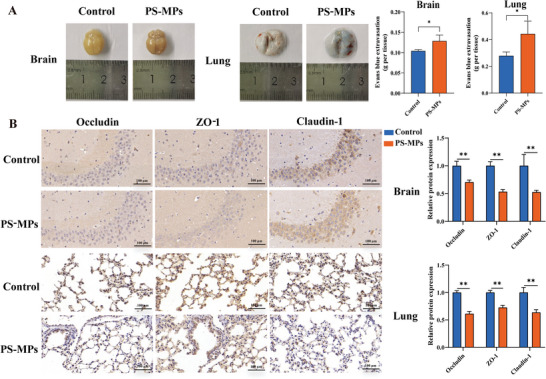
The damage of PS‐MPs to blood‐brain barrier and air‐blood barrier. A) The damage of barrier function. B) Tight junction protein expression in brain and lung. **p < 0.01.

The integrity of BBB and air‐blood barrier (ABB) was further examined by intravenous Evans blue injection and both were found to be impaired (**Figure** **3**A) and tight junction protein (TJ) expression was decreased (Figure 3B). However, the cause of barrier dysfunction and the mechanism by which it affects nervous system function through the lung‐brain axis are still unclear.

### 2.4 Pulmonary Flora/LPS Mediated Lung‐Brain Axis is Involved in PS‐MPs‐Induced Neurotoxicity

**Figure 4:**
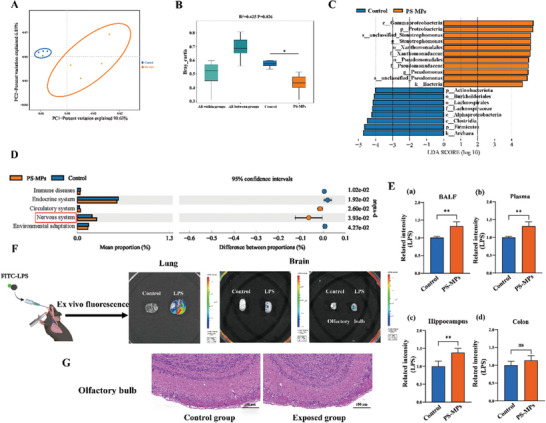
Pulmonary flora and LPS expression in mice after inhalation of PS‐MPs. A) PCA plot analysis (*n* = 4). B) Anosim analysis. C) Differential pulmonary flora identification by LEfSe analysis. D) Differential analysis of KEGG metabolic pathways. E) Expression of LPS in BALF, Plasma, hippocampus, and Colon. F) Distribution of LPS in the lung and brain. G) Histopathological damage of the Olfactory bulb. ^ns^ *p* > 0.05, **p < 0.01. BALF: bronchoalveolar lavage fluid.

Studies have found that respiratory exposure to MPs can cause microbiota imbalance in the nose and lung of mice.^[^ ^26^, ^27^ ^]^ Specifically, a total of 800,997 read pairs were obtained from all BALF samples, and 693,058 clean reads were generated after quality control and splicing of the paired‐end reads. Each sample generated at least 62,783 clean reads, with an average of 86,632 clean reads. The Shannon index was used to assess microbial diversity in the samples. The flattening of the Shannon index curve indicated that the amount of microbial sequencing data in this study was sufficient. This suggests that the read depth and quality of the sequences obtained in this study were high (Figure S3A, Supporting Information). Our study also found that PS‐MPs exposure caused bacterial dysbiosis in the lungs of mice, with gram‐negative bacteria becoming the dominant genus (**Figure** **4**A–C; Figure S3B,C, Supporting Information). KEGG enrichment analysis showed that changes in pulmonary flora may affect the function of the nervous system (Figure 4D). Interestingly, the transcriptomic results of lung tissues in this study also found that Toll‐like receptor signaling pathway was activated. Notably, LPS as a component of the cell wall of gram‐negative bacteria and an important activator of Toll‐like receptor 4 (TLR4) has attracted our attention. LPS was detected in BALF, plasma, hippocampus, and colon. We found that LPS was increased in all samples except the colon (Figure 4E). Next, we observed the distribution of FITC‐LPS in the body by intratracheal instillation for 8 w. LPS accumulation was found in both lung and brain tissue. The olfactory bulb is the site of LPS entering brain tissue (Figure 4F). In addition, we found that mice exhibited a reduced number of periglomerular cells around the synaptic glomerular layer and disturbances in the granular cell layer of olfactory bulb tissue (Figure 4G), further supporting this process. These results suggest that the increase in LPS, driven by MP‐induced alterations in the pulmonary flora, may enter the brain via the olfactory bulb and contribute to nerve damage.

**Figure 5:**
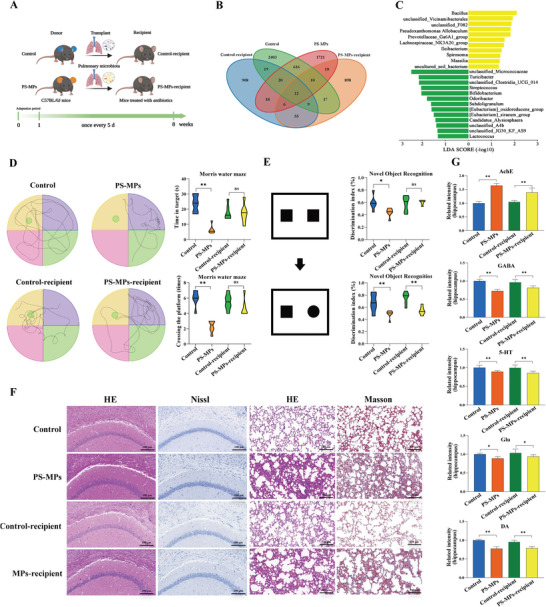
Effect of pulmonary flora transplantation (PFT) on nerve injury. A) Schematic diagram of the pulmonary flora transplantation process. B) Distribution of OTUs in the four groups. C) The changes of pulmonary flora after PFT were analyzed by metastats analysis. D) Results of morris water maze test (*n* = 6). E) Results of novel object recognition test (*n* = 6). F) Histopathological findings of the brain and lung. G) Expression of important neuroplasticity‐related molecules in the hippocampus (*n* = 5). ^ns^ *p* > 0.05, *p < 0.05, **p < 0.01.

To verify the role of pulmonary flora in PS‐MPs‐induced nerve injury, we performed PFT experiments (**Figure** **5**A). Antibiotic clearance did not affect body weight (Figure S4A, Supporting Information). The composition and abundance of pulmonary flora in the BALF were detected at 8 w (Figure 5B; Figure S4B–E, Supporting Information). Compared with the Control‐recipient group, the pulmonary flora of PS‐MPs‐recipient group showed an increasing trend of gram‐negative bacteria (Figure 5C). The results of behavioral experiments showed that there was no significant difference in the number of crossing the platform and the time of staying in the target in the MWM test between the two groups (Figure 5D). The escape latency, total swim distance, and swim speed during training are shown in Figure S4G (Supporting Information). In the NOR test, compared with the Control‐recipient group, the PS‐MPs‐recipient group showed a decreased recognition index of the novel object (Figure 5E). Histopathological results showed that the antibiotic clearance process had no obvious damage to the lung tissue of mice and had no effect on the subsequent experiments (Figure S4F, Supporting Information). There were no significant changes in brain tissue and lung tissue between the Control‐recipient group and the Control group. The brain tissue and lung tissue of PS‐MPs‐recipient mice showed similar damage to PS‐MPs group, but the degree of damage was relatively mild (Figure 5F). Compared with the Control‐recipient group, the expression of AChE was increased and the levels of GABA, 5‐HT, Glu, and DA were decreased in the plasma and hippocampus of the PS‐MPs‐recipient group (Figure 5G; Figure S4H, Supporting Information). Pulmonary flora plays an important role in the process of nerve injury induced by PS‐MPs, and the increased abundance of gram‐negative bacteria is one of the important reasons.

**Figure 6:**
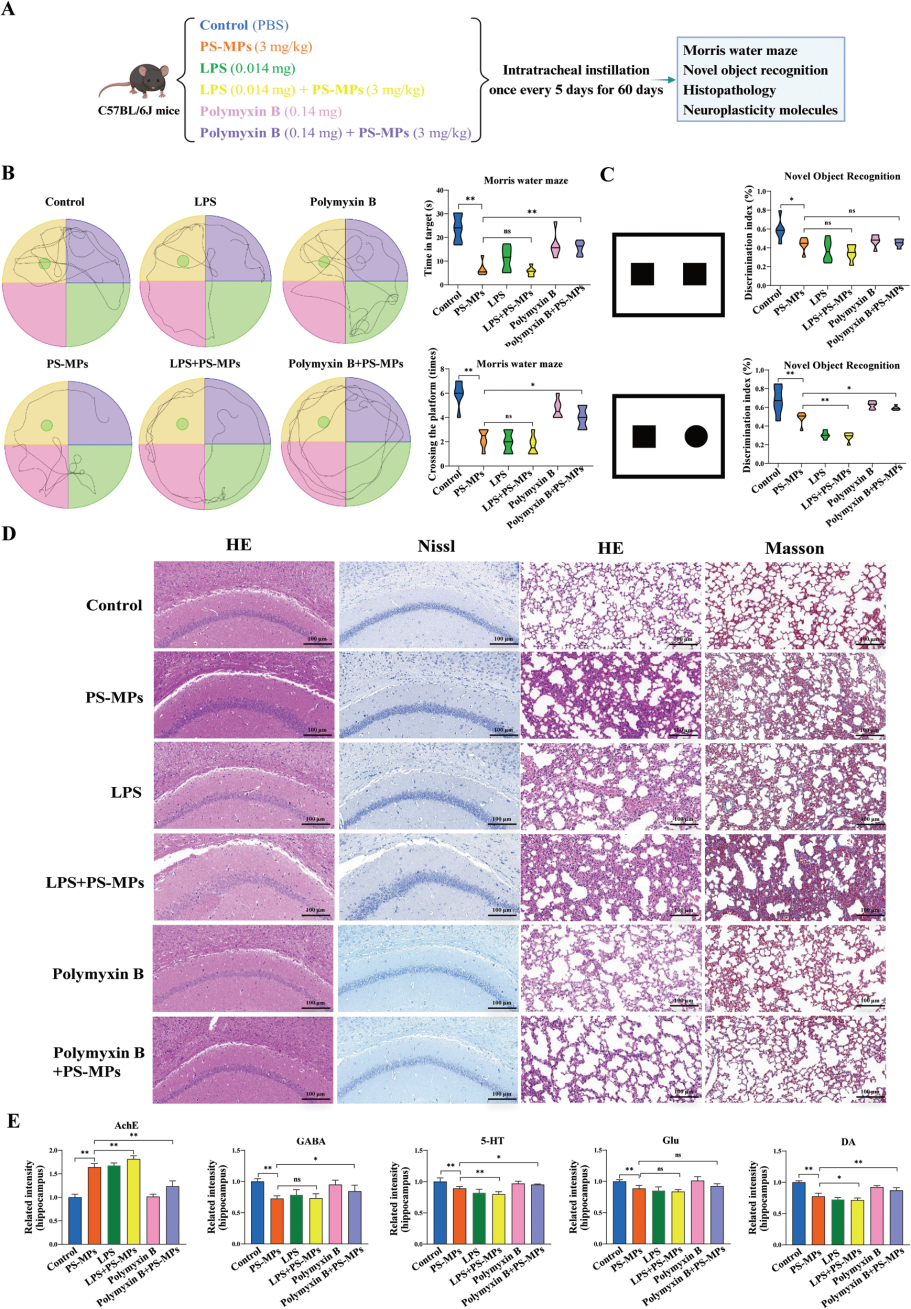
Effects of LPS and LPS inhibitor (polymyxin B) intervention on nerve injury. A) Schematic illustration of the intervention process. B) Results of morris water maze test (*n* = 6). C) Results of novel object recognition test (*n* = 6). D) Histopathological findings of the brain and lung. E) Expression of important neuroplasticity‐related molecules in the hippocampus (*n* = 5). ^ns^ *p* > 0.05, *p < 0.05, **p < 0.01.

LPS is an endotoxin produced by gram‐negative bacteria, which can damage the BBB function and further promote the occurrence and development of nervous system diseases.^[^ ^33^ ^]^ In order to explore the role of LPS in PS‐MPs‐induced nerve injury, we used LPS and polymyxin B (LPS inhibitor) for intervention experiments (**Figure** **6**A). The results of behavioral experiments showed that compared with the PS‐MPs group, the LPS+PS‐MPs group had no significant difference in the number of platform crossings and the time spent in the target quadrant in the MWM test (Figure 6B). The escape latency, total swim distance, and swim speed during training are shown in Figure S5A (Supporting Information). In the NOR test, the recognition index of the novel object was decreased in mice (Figure 6C). Compared with the PS‐MPs group, the polymyxin B+PS‐MPs group had a significantly increased number of platform crossings and time spent in the target quadrant in the MWM test (Figure 6B). In the NOR test, the recognition index of the novel object increased in mice (Figure 6C). Histopathological results showed that LPS increased the damage of brain tissue and lung tissue in mice, while Polymyxin B alleviated the damage of both (Figure 6D). Compared with the PS‐MPs group, LPS+PS‐MPs mice showed increased AChE level in hippocampus, decreased 5‐HT and DA levels in plasma and hippocampus, and decreased Glu level in plasma. Polymyxin B+PS‐MPs mice showed increased levels of AChE and decreased levels of GABA, 5‐HT, Glu, and DA in plasma and hippocampus (Figure 6E; Figure S5B, Supporting Information). Together, pulmonary flora/LPS mediated lung‐brain axis is one of the important mechanisms of nerve injury induced by PS‐MPs.

### 2.5 LPS Causes Neurotoxicity by Causing M1 Polarization of Microglia

**Figure 7:**
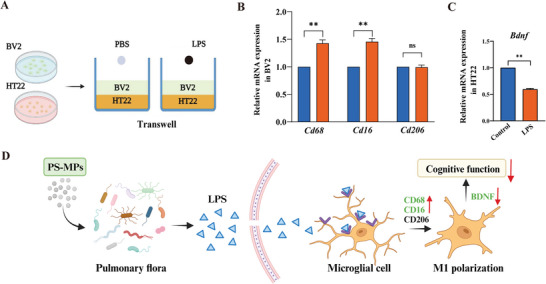
Effect of LPS on microglia and hippocampal neurons (*n* = 5). A) Schematic diagram of cell co‐culture and intervention. B) Activation of BV2 cells. C) Expression of *Bdnf* in HT22 cells. D) Schematic representation of impaired cognitive function after inhalation of PS‐MPs. ^ns^ *p* > 0.05, **p < 0.01. LPS: Lipopolysaccharide; *Bdnf*: brain‐derived neurotrophic factor.

The process by which inhaled PS‐MPs affect cognitive function in mice through an increase in LPS levels remains unclear. Microglia are innate immune effector cells in the central nervous system, which contribute to both inflammatory damage and tissue repair in nervous system diseases.^[^ ^34^ ^]^ We established a co‐culture system of mouse microglia (BV2) and hippocampal neurons (HT22) to investigate the specific mechanism of nervous system injury caused by PS‐MPs through LPS increase (**Figure** **7**A). We selected the optimal dose of LPS (1 µg mL^−1^) by observing cell viability (Figure S6, Supporting Information). We found that LPS induced M1 polarization of BV2 cells (Figure 7B) and increased brain‐derived neurotrophic factor (BDNF) mRNA expression in HT22 cells (Figure 7C). These results suggest that PS‐MPs can induce M1 polarization of microglia through the changes of pulmonary flora to produce more LPS, and then affect the function of the nervous system (Figure 7D).

## 3 Discussion

Researchers have detected MPs in drinking water,^[^ ^35^ ^]^ food,^[^ ^36^ ^]^ and air.^[^ ^7^, ^37^ ^]^ Humans can be exposed to MPs through ingestion, inhalation, and dermal contact. PS‐MPs can induce toxic effects on multiple organs and systems, including digestive, respiratory, nervous, reproductive, and cardiovascular systems.^[^ ^38^ ^]^ Current research primarily focuses on the hazards caused by MPs exposure in the digestive tract. However, compared to the digestive tract, the intake of MPs through the respiratory tract is characterized by prolonged exposure due to continuous respiratory movement, encompassing multiple exposures to both indoor and outdoor air. More importantly, studies have confirmed the presence of MPs in the air,^[^ ^5^, ^39^ ^]^ human lung tissue,^[^ ^1^, ^2^ ^]^ and nasal cavity.^[^ ^3^, ^4^ ^]^ Therefore, the health hazards of inhaled MPs on human populations require further study and exploration.

Air pollution is among the most significant environmental health risks. A growing body of epidemiological studies has linked air pollution to various neurodegenerative diseases and other forms of nervous system damage, findings that are supported by animal experiments.^[^ ^40^ ^]^ According to World Health Organization statistics, neurotoxic environmental pollutants and other risk factors have contributed to neurological diseases in more than 3 billion people worldwide as of 2021. The total number of disabilities, illnesses, and premature deaths due to these neurological conditions has increased by 18.2% since 1990.^[^ ^41^, ^42^ ^]^ Up to 84% of disability‐adjusted life years lost to stroke could be prevented by controlling high systolic blood pressure and reducing both ambient and household air pollution.^[^ ^43^ ^]^ Recent studies have shown that the intake of MPs can cause nervous system damage^[^ ^44^, ^45^ ^]^ and even impair the cognitive function^[^ ^46^, ^47^ ^]^ in mice, as demonstrated through both in vitro and in vivo experiments. These findings suggest that MPs should also be classified as neurotoxic environmental pollutants.

As an emerging pollutant, the health impacts of MPs have been increasingly studied. Current research indicates that smaller MPs are more harmful to the body.^[^ ^48^ ^]^ Consequently, many studies have focused on smaller particle‐size nanoplastics (NPs) as their research subjects. However, sources of airborne MPs include plastic particles added to industrial products and those formed through the decomposition and volume reduction of large plastic waste via physical, chemical, and biological processes. Researchers found that 87.5% of the total MPs in the human lung were microsized, with a particle size range of 1.60‐5.56 µm.^[^ ^2^ ^]^ This suggests that research on NPs may not fully represent real environmental exposure in humans, and further exploration of the specific mechanisms is needed. In our study, 5 µm PS‐MPs was selected for experimental, as this size closely matches the internal exposure to MPs under real conditions and is similar to PM_2.5_. Previous studies have found that PM_2.5_ may affect the central nervous system function by disrupting the BBB,^[^ ^49^ ^]^ causing neuroinflammation,^[^ ^50^ ^]^ and influencing the gut‐brain axis.^[^ ^51^, ^52^ ^]^ These findings provide insights relevant to our study, but the specific mechanisms of nervous system damage caused by inhaled MPs require further investigation.

Currently, there is no evidence of MPs entering human brain tissue, although toxicological studies have shown that NPs ranging from 50 to 500 nm can penetrate adult animal brain tissue.^[^ ^12^, ^47^, ^53^ ^]^ However, it has been found that 5 µm MPs ingested through the digestive tract can enter the brains of aging mice.^[^ ^53^ ^]^ This suggests that particle size and age may influence the direct effects of MPs on an organism. In our study, we found that while micron‐scale 5 µm PS‐MPs did not accumulate in brain tissue following inhalation, they impaired the ABB and BBB of mice and decreased cognitive function. In addition, PS‐MPs can indirectly cause nerve damage through various substances and pathways. Studies have found that MPs cause cognitive impairment by inducing oxidative stress,^[^ ^55^ ^]^ neuroinflammation,^[^ ^47^ ^]^ disrupting energy metabolism,^[^ ^56^ ^]^ arresting the cell cycle, and accelerating brain aging.^[^ ^57^ ^]^ Interestingly, previous studies have found that inhaled MPs are more likely to pass through the “airborne microbiomes‐lung” axis and cause lung injury compared with consumed MPs.^[^ ^27^ ^]^ Chronic obstructive pulmonary disease (COPD) is known to directly and independently increase the risk of stroke by causing systemic inflammation and increasing oxidative stress, thereby promoting cerebrovascular dysfunction.^[^ ^58^ ^]^ One study found that patients with COPD or restricted lung function exhibited more lacunar infarcts and poorer overall cognitive function.^[^ ^59^ ^]^ There is a strong link between pulmonary flora and brain immune reactivity.^[^ ^21^ ^]^ Therefore, pulmonary changes induced by inhalation of PS‐MPs may contribute to the development of cognitive impairment.

Alexander Flugel et al.^[^ ^21^ ^]^ published a paper in *Nature* that revealed the existence of the lung‐brain axis. Briefly, pulmonary flora can modulate brain immune responses by producing LPS. The adaptive immune system driven by microglia can affect the cognitive function of the brain. Interestingly, we also found that MPs inhalation disturbed the pulmonary flora in mice, specifically leading to an increase in the abundance of gram‐negative bacteria. Gram‐negative bacteria are associated with neuropathology in Alzheimer's disease (AD), with LPS considered the most potent microbial mediator, indicating a threat from gram‐negative bacterial invasion. LPS co‐localized with Aβ_1‐40/42_ in amyloid plaques and perivascular areas of the AD brain.^[^ ^60^ ^]^ LPS levels in the brain were also higher in AD patients than in controls.^[^ ^61^ ^]^ In our study, LPS levels were increased in BALF, hippocampus, and plasma of PS‐MPs mice, but not in the colon. Therefore, the LPS increase caused by pulmonary flora disturbance is a key factor in the cognitive impairment induced by PS‐MPs. In addition, microglia are key nervous system‐specific immune cells that can influence brain development, maintenance of the neural environment, response to injury, and repair.^[^ ^62^ ^]^ LPS can stimulate microglia to polarize into the M1 phenotype, increasing the expression of pro‐inflammatory factors. This process is typically accompanied by a shift from oxidative phosphorylation to aerobic glycolysis for energy production, promoting the onset and progression of neurodegenerative diseases.^[^ ^63^ ^]^ The lung‐brain axis is a crucial mechanism underlying cognitive dysfunction induced by PS‐MPs inhalation in mice, though the specific processes have not been previously reported.

In our study, PFT assays were used to exclude other confounding factors, revealing that the disturbance of pulmonary flora caused by inhalation of PS‐MPs impaired cognitive function in mice and increased the proportion of gram‐negative bacteria in the pulmonary flora. We further examined the role of LPS using intervention experiments with LPS and its inhibitor, polymyxin B, finding that intratracheal instillation of LPS produced neurological deficits similar to those induced by inhalation of PS‐MPs, while treatment with polymyxin B alleviated these deficits. This demonstrates the involvement of LPS as a mediator in PS‐MPs‐induced nerve injury. In vitro co‐culture experiments were conducted to explore the potential mechanisms by which PS‐MPs affect cognitive function. BDNF is closely associated with the survival, maintenance, and regeneration of specific neuron populations in the brain. Microglia can regulate neuroplasticity via BDNF, thereby affecting cognitive function.^[^ ^64^ ^]^ Our study found that LPS stimulates M1 polarization of microglia and inhibits BDNF expression in hippocampal neurons, identifying it as a key mechanism underlying cognitive dysfunction following PS‐MPs inhalation in mice.

## 4 Limitations of the Study

It is important to acknowledge several additional limitations in this study. (1) The study was limited to 5 µm PS‐MPs. The types, sizes, and shapes of MPs in the real environment are highly complex and difficult to simulate. Different MPs may pose varying health hazards. Our study provides only a preliminary exploration of MPs‐induced neurotoxicity. (2) The method of intratracheal instillation cannot fully replicate the natural respiratory inhalation process. In future studies, we will utilize a broader range of respiratory exposure methods to establish animal models that more accurately reflect the toxic effects of MPs. (3) Pulmonary flora can vary between different animal species. Our study was conducted solely on C57BL/6J mice, a common model organism, which presents certain limitations. In future research, we will establish a variety of animal models to explore the differences in pulmonary flora across species. (4) The organism is a complex biological system, and the development of disease or injury often involves multiple mechanisms. Our study has uncovered only one of the mechanisms by which inhalation of MPs cause nerve damage. Further research is needed to explore additional mechanisms and provide a more comprehensive explanation of the specific causes of nerve damage.

## 5 Conclusion

In summary, our study examines pulmonary flora, immune response, and neuroplasticity, demonstrating that cognitive function is closely linked to lung health. We found that inhalation of PS‐MPs promotes M1 polarization of microglia and impairs cognitive function in mice by altering pulmonary flora to increase LPS production. In brief, pulmonary flora‐derived lipopolysaccharide mediates lung‐brain axis through activating microglia, which is one of the mechanisms behind cognitive dysfunction induced by inhalation of PS‐MPs. Inhibiting LPS may offer a preventive strategy against nerve injury caused by inhalation of MPs. This study offers a new perspective on the neurotoxicity of PS‐MPs through multi‐organ interaction, suggests new preventive measures for nervous system damage caused by environmental pollutants, and lays the groundwork for future research. In the future, we anticipate more research on the role of the lung‐brain axis in neurotoxicity caused by environmental pollutants, with the goal of developing more effective strategies to reduce nervous system damage in the population.

## 6 Experimental Section

### Chemicals

Studies based on human lung samples found that 87.5% of MPs were at the micron scale, with particle sizes ranging from 1.60 µm to 5.56 µm.^[^ ^2^ ^]^ This indicates that micron level MPs were significant components of internal exposure. MPs of 5 µm was selected to simulate exposure in a real environment.

The morphology of 5 µm PS‐MPs (Baseline Chromatography Technology Development Center, China) were observed by scanning electron microscopy (SEM, Hitachi S‐4800, Japan, resolution = 1 nm, accelerating voltage = 3.0 kV, detector: low/high secondary electron detector).^[^ ^48^ ^]^ The hydrodynamic size and zeta potential of PS‐MPs were measured using Nano ZSP Expert Colloid & Protein (Malvern, UK) at 25 °C, with a concentration of 50 µg mL^−1^ and PH7.4.^[^ ^48^ ^]^

### Animals and Experimental Design

C57BL/6J male mice (Six‐week‐old, Vital River Laboratory Animal Technology, China) were housed in a standard SPF environment, with a 12 hours (h) light/dark cycle, a temperature of 22 ± 2 °C, and humidity maintained at 50–60%. The mice were fed ad libitum with sterilized rodent chow and autoclaved water. All experiments in this study were approved by the Capital Medical University Animal Care and Use Committee (AEEI‐2020–168). Based on previous studies,^[^ ^6^, ^65^ ^]^ 0.6 mg kg^−1^ was chosen as the exposure dose for mice. The formulas for the calculation of PS‐MPs exposure were provided in the Supporting Information. Considering that age and work environment were important factors affecting MPs intake.^[^ ^66^ ^]^ To simulate the exposure of different exposed populations, a 5‐fold concentration gradient was designed. Mice were randomly divided into four groups (*n* = 8). After 7 days of acclimatization, 50 µL PBS containing 0.6, 3, and 15 mg kg^−1^ PS‐MPs (PS‐MPs group) or an equal volume of PBS (Control group) were administered via intratracheal instillation every 5 d (detailed methods were provided in the Supporting Information). After 60 d of experimental treatment, the mice were euthanized with 0.3% pentobarbital sodium administered intraperitoneally, followed by PBS perfusion. Tissues were harvested, sectioned for histopathological examination, and fixed in 4% paraformaldehyde (PFA) or 2.5% glutaraldehyde. The remaining tissue samples were immediately frozen in liquid nitrogen and stored at ‐80 °C.

### PFT Experiment

The experiment contains the following four groups: Control group, PS‐MPs group, Control‐recipient group, and PS‐MPs‐recipient group. Antibiotics (10 µg ciprofloxacin + 50 µg metronidazole, Sigma‐Aldrich, USA) were instillation into the trachea to eliminate the original bacteria in the lungs of the mice, once every 3 d for 5 times. The Control group and PS‐MPs group were exposed to PBS and PS‐MPs (3 mg kg^−1^) every 5 d, and then the pulmonary flora was transplanted to the Control‐recipient group and PS‐MPs‐recipient group at 2, 4, 6, and 8 w, respectively. The procedure was as follows: BALF (450 µL per mouse) was collected from the donor group, BALF was centrifuged at 13,000 rpm for 10 minutes (min) at 4 °C, and the pulmonary flora precipitate was resuspended in 150 µL of sterile PBS.^[^ ^21^ ^]^ Each mouse in the recipient group received 50 µL of the corresponding resuspension via intratracheal instillation every 5 d. The BALF from mice in the same donor group was pooled and then transplanted to ensure consistency in the species and quantity of bacteria received by each mouse in the recipient group.

### LPS Intervention Experiment

The experiment contains the following six groups: Control group, PS‐MPs group, LPS group, LPS+PS‐MPs group, polymyxin B, and polymyxin B+PS‐MPs group. The exposure methods of the Control group and PS‐MPs group were the same as above. 0.014 mg LPS^[^ ^21^ ^]^ or 0.14 mg polymyxin B^[^ ^21^ ^]^ was intratracheally instilled in the LPS group and polymyxin B group every 5 d for 60 days. The LPS+PS‐MPs group or polymyxin B+PS‐MPs group was intratracheal instilled PS‐MPs 2 h after LPS or polymyxin B exposure.

### Cell Culture and Experimental Design

Murine BV2 microglia cells were cultured in DMEM‐H medium with 10% fetal bovine serum (Gibio, USA), and 1% Penicillin‐Streptomycin solution (Biosharp, China) in a 37 °C and 5% CO_2_ humidified incubator. BV2 cells were treated with LPS (0, 0.2,^[^ ^67^ ^]^ 1,^[^ ^68^ ^]^ and 5 µg mL^−1^) for 24 h.

Mouse hippocampal neuronal cells (HT22) were cultured under the same conditions as BV2 cells. BV2 cells and HT22 cells were cultured into transwell chambers (0.4 µm, Corning, costar‐3450, USA) and six‐well plates in advance to allow enough time to adhere to the wall, and the cell density reached more than 50% after 24 h of adherence. As shown in Figure 7A, BV2 cells were subjected to indirect contact serum‐free co‐culture with HT22 cells for 24 h.

### Behavioral Experiment

*Morris Water Maze (MWM) Test*: The MWM test was used to detect spatial learning and memory abilities in mice. The experimental procedure was consistent with a previous study.^[^ ^69^ ^]^ A circular plastic platform (8 cm in diameter, hidden 1 cm beneath opaque water) was placed in one of the four quadrants of a water maze pool with a diameter of 120 cm and an inner wall height of 60 cm (22 ± 2 °C, <60 dB, <100 lux). Mice were placed in the water maze laboratory for 1 h before the experiment to acclimate to the environment and reduce stress. They underwent three trials per day for five consecutive days. The mice were allowed 1 min to search for the platform in each trial.

*Novel Object Recognition (NOR) Test*: NOR test was used to evaluate the non‐spatial learning and memory ability of mice. Mice were placed in the testing room (22 ± 2 °C, humidity at 50–60%, < 60 dB, 15 lux) to acclimate 1 h before the sessions.^[^ ^47^ ^]^ All experiments were performed in the same white test chamber (50 cm × 50 cm × 40 cm) at the same time of day. On day 1, two identical objects, A and B (cube, wood, 3 cm × 3 cm × 3 cm), were placed in the test chamber and allowed to move freely for 5 min.^[^ ^70^ ^]^ On day 2, object B was replaced with object C (cylinder, wood, diameter = 3 cm, height = 3 cm), which was identical to object A except for its different shape and material, and allowed to move freely for 5 min. Mice were observed to see if they had a preference for two objects of different shapes. The exploration time of mice for object A was recorded as F (s), and the exploration time for objects B and C was recorded as N (s). The discrimination index (DI) was calculated as equation (1).

(1) $$\mathrm{DI}\;\;\left(\%\right)=\frac{N}{\left(N+F\right)}\;\times 100\%$$

*Three‐Chamber Sociability Test*: The three‐box social test was used to measure the social behavior of the animals. The sociability of the animals was determined by comparing the time spent near the metal cage of familiar mice with the time spent near the metal cage of unfamiliar mice. The three‐chamber box was a rectangular device (45 cm × 15 cm × 40 cm) with a 15 cm partition between each chamber. First, experimental mice were placed in the middle chamber. Then, open the divider on both sides of the round wire cup chamber, so that it can enter the two chambers at will. Strange mice (S1 and S2) had the same background, age, sex, and weight as the experimental mice. They had never met before the experiment. One cup contained a strange mice (S1) and the other was empty (E). Experimental mice were allowed to explore the cup and the entire apparatus for 10 min.^[^ ^47^ ^]^ Finally, empty cups (E) were replaced with new mice (S2), and then the experimental mice were allowed to explore again for 10 min. The sniffing time between S1, E, and S2 was observed.

*Data Collection and Analysis*: The video camera was used to record the process of mice in the whole experiment, and Tracking master v3.0 software^[^ ^69^ ^]^ was used for data analysis.

### Histopathology

After 60 d of PS‐MPs exposure, brain and lung tissues were fixed in 4% PFA. The rest of the procedure was the same as in the previous experiment.^[^ ^69^ ^]^ In brief, paraffin embedding was followed by sectioning at a thickness of 4 µm. Three animals were randomly selected from each group, and one slide was prepared from each animal. H&E staining, Nissl, and Masson staining were used to compare the histomorphological differences among the groups. High‐resolution images were obtained by panoramic scan (3DHISTECH, Hungary, HPF, × 200 magnification) analyzed by Case Viewer software. All slides were analyzed by a single researcher.

### Transmission Electron Microscopy

Hippocampus and lung tissues (1 mm^3^) were fixed in 2.5% glutaraldehyde for 24 h and stored at 4 °C. After penetrating embedding (acetone: 812 embedding agent = 1:1 at 37 °C for 2–4 h, followed by acetone: 812 embedding agent = 1:2 at 37 °C overnight, and finally pure 812 embedding agent at 37 °C for 5–8 h), ultrathin sections (60–80 nm) were prepared. The sections were stained with 2% uranyl acetate for 8 minutes and 2.6% lead citrate for 8 min, then observed using transmission electron microscopy (Hitachi HT7800, Japan; resolution = 1 nm, accelerating voltage = 4.0 kV), and images were collected.

### RNA‐Sequencing

Total RNA was extracted from mouse hippocampus and lung tissues using Trizol reagent, following previously described methods.^[^ ^71^ ^]^ The rest of the procedure was the same as in the previous experiment.^[^ ^69^ ^]^ Briefly, mRNA was purified from total RNA using poly‐T oligo‐attached. Hierarchical clustering of differentially expressed mRNAs was performed (R package pheatmap, vension: 1.0.12). KEGG enrichment of differentially expressed genes was performed (FDR < 0.05, FC > 1.2), and the significance was set at *p* < 0.05.

### ELISA

The levels of acetylcholinesterase (AChE), γ‐aminobutyric acid (GABA), serotonin (5‐HT), glutamate (Glu), and dopamine (DA) in the hippocampus or plasma. The levels of LPS in BALF, plasma, hippocampus, and colon were measured by ELISA kits (Jiangsu Jingmei Biological Technology Co., Ltd., China). Standard curves and detection limits for kits in the experiment were shown in the Table S1 (Supporting Information).

### Evaluation of Barrier Permeability

Evans blue dye of 2% (2 mL kg^−1^) was injected into the tail vein and allowed to circulate for 15 min, followed by transcardial perfusion with PBS until the fluid in the right atrium was colorless. The brain and lung tissues were then separated and photographed for comparative observation. The dye was subsequently extracted with 1 mL formamide (Sigma‐Aldrich) overnight at 50 °C. Whole tissues were dried for 1 h before being weighed. The concentration of Evans blue in formamide extracts at 620 nm was determined using a spectrophotometer (Tecan Spark, Austria), using formamide as a blank reference. The Evans blue dye content was calculated by normalizing the extracted dye quantity to the tissue weight.

### Fluorescence Imaging

Mice were exposed to fluorescent PS‐MPs (5 µm red fluorescent labels, Ex/Em: 620/680 nm) or 0.014 mg FITC‐LPS (Sigma‐Aldrich, USA) for 8 w. Based on previous studies,^[^ ^72^ ^]^ live mice or fresh tissues were placed on the sample tray for fluorescence imaging 24 h after the last exposure. The distribution of PS‐MPs in lung and brain tissues was traced using an IVIS®Lumina LT Series III imaging system (PerkinElmer Ltd, USA) with excitation at 605 nm and emission at 680 nm. The influence of autofluorescence was eliminated by comparison between groups.

### Immunohistochemistry

The brain and lung tissue sections were incubated with primary antibodies including, Occludin, Claudin‐1, zonula occludens‐1 (ZO‐1) (1: 500, Affinity, USA, Goat Anti‐Rabbit IgG) overnight (4 °C), and were then incubated with horse radish peroxide‐conjugated secondary antibodies (1: 500, Merck Millipore, Germany) and 3, 3‐diaminobenzidine substrate (Sigma‐Aldrich, USA). High‐resolution images were obtained by panoramic scan (3DHISTECH, Hungary, × 200 magnification) analyzed by Case Viewer software. Qualitative and quantitative evaluation criteria for staining were followed previously described methods.^[^ ^73^ ^]^

### Pulmonary Flora Analysis

Total genomic DNA from the BALF (dissolved in PBS and pre‐stored at −80 °C) was extracted using the Synergy HTX Multi Mode Reader (Gene Company Ltd., China). The rest of the procedure was the same as in the previous experiment.^[^ ^65^ ^]^ Further sequencing was performed on the PacBio sequencing platform (Biomarker Technologies or BMKcloud, Beijing, China). The Alpha diversity index (Chao1 index and Shannon index) of the samples was evaluated by QIIME2 2020.6 software. The diversity of species in the samples was assessed using Principal Component Analysis (PC1 and PC2, Oval circles indicate the range in which 95% of the sample was present), R software was used for visual analysis of the results. Differences in species abundance between groups were analyzed using Metastats software. The *t*‐test was used to analyze species abundance data between groups, *p* < 0.05 determined statistical significance.

### Cell Viability Assay

The experimental procedure was consistent with the previous study.^[^ ^74^ ^]^ BV2 cells were seeded in 96‐well plates and set as a blank group, control group, and LPS group (0, 0.2, 1, and 5 µg mL^−1^). After 24 h of incubation, 10 µL CCK‐8 solution (Biorigin, China) was added to each well and incubated for another 2 h. Finally, the absorbance was measured in a microplate reader (450 nm).

### Real‐Time Quantitative PCR (RT‐qPCR):

Total RNA from brain, lung, HT22 cells, and BV2 cells was extracted with Trizol. cDNA from the samples was obtained using cDNA reverse transcription kit (Allmeek, China). RT‐qPCR analysis was performed using the qPCR kit (Allmeek, China) in Bio‐Rad. The 2^−ΔΔCT^ method was adopted to calculate the tested genes which were normalized by β‐actin.^[^ ^75^ ^]^ The primer sequence was shown in Table S2 (Supporting Information).

### Statistical Analysis

All statistical parameters were expressed as mean ± standard deviation (SD). Data were tested for homogeneity using Bartlett's test for unequal variances and for normality using the Shapiro‐Wilks *W*‐test. A two‐tailed unpaired Student's *t*‐test was used to compare the significance between two groups. One‐way ANOVA followed by Tukey's multiple comparison test with Bonferroni correction was used to determine the significance of multiple group comparisons. Data that did not conform to normality were compared between multiple groups using the Games‐Howell test. Data analysis was performed using SPSS 26.0 or GraphPad 8.3 software.

## Conflict of Interest

The authors declare no conflict of interest. Figures were created with biorender.com.

## Author Contributions

H.K. performed experiments, statistical analysis and drafting of the manuscript; D.H. study concept and design, performed experiments. W.Z., J.W., and Z.L. performed experiments; Z.W., and G.J. acquisition of data and drafting of the review; A.G. study concept and design, obtained funding.

## Supporting information



Supporting Information

## Data Availability

The data that support the findings of this study are available from the corresponding author upon reasonable request.
