# Supplementary material for: Pulmonary Flora‐Derived Lipopolysaccharide Mediates Lung‐Brain Axis through Activating Microglia Involved in Polystyrene Microplastic‐Induced Cognitive Dysfunction
Source: Adv Sci (Weinh). 2024 Nov 5;11(47):2404966. doi: 10.1002/advs.202404966 (PMC11653722; doi:10.1002/advs.202404966)
Supplement: Supplementary file 1 — Supporting Information [file ADVS-11-2404966-s001.docx]

**Supporting Information**

**The specific operation process of intratracheal instillation:**

After administering intraperitoneal anesthesia with pentobarbital sodium, an indwelling needle was placed into the glottic detrusor of the mice, and 50 μL of liquid was injected through the needle. The mice were then placed on a constant temperature heating plate and returned to their original cages after recovery.

**The PS-MPs exposure was calculated using the following formulas:**

(1) The weight of the largest size of inhalable particles (10 μm) PS-MPs.

m=ρV=1.05 g cm^-3^×$\frac{4}{3}\times\pi({\frac{10 \mu m}{2})}^{3}$=5.49×10^-7^ mg

(2) Quality of daily PS-MPs exposure in Chinese adults.

5.49×10^-7^ mg×490 MPs m^-3^×15.7 m^3^ day^-1^=4 μg d^-1^

(3) Quality of PS-MPs exposure per 5 days for one mouse.

4 μg d^-1^×5 d÷30 g≈0.6 mg kg^-1^

**Figure S1. Supplementary material for nerve injury induced by inhalation of PS-MPs.** (A-B) Hydrodynamic size of PS-MPs. (C) Body weight of mice. (D) Supplementary parameters for the morris water maze. (E) Three-chamber sociability test was used to detect the social ability of mice (n = 6). (F) Expression of important neuroplasticity-related molecules in differentially expressed signaling pathways in the plasma (n = 5). ns p > 0.05, * p < 0.05, ** p < 0.01.

**Figure S2. Specific distribution and quantitative analysis of fluorescent PS-MPs in various tissues of the body (n = 3).** (A) The distribution of PS-MPs. (B) Quantification of PS-MPs in various tissues. ^ns^ p > 0.05, ** p < 0.01.

**Figure S3. Supplementary material for the pulmonary flora.** (A) Shannon index curve. (B) Chao 1 index. (C) Shannon index. ** p < 0.01.

**Figure S4. Supplementary material for the pulmonary flora transplantation.** (A) Body weight of mice. (B) Chao 1 index. (C) Shannon index. (D) NMDS analysis. (E) Anosim analysis. (F) Histopathological damage of brain and lung tissues in mice treated with antibiotics. (G) Supplementary parameters for the morris water maze. (H) Expression of neuroplasticity-related molecules in plasma after pulmonary flora transplantation (n = 5). ^ns^ p > 0.05, * p < 0.05, ** p < 0.01.

**Figure S5. Supplementary material for the LPS intervention experiment.** (A) Supplementary parameters for the morris water maze. (B) Expression of neuroplasticity-related molecules in plasma after LPS intervention (n = 5). ^ns^ p > 0.05, ** p < 0.01.

**Figure S6. Effect of different concentrations of LPS on the viability of BV2 cells (n = 5).** ^a^ P < 0.05, compared with the 0 μg mL^-1^ group; ^b^ P < 0.05, compared with the 0.2 μg mL^-1^ group; ^c^ P < 0.05, compared with the 1 μg mL^-1^ group.

**Table S1 The** **standard curves and detection limits for ELISA kits**

| Name | Standard curve | R^2^ | Detection limit |
| --- | --- | --- | --- |
| AChE | Y=0.0021X | 0.9995 | 0.1 nmol L^-1^ |
| GABA | Y=0.1773X | 0.9991 | 0.1 ng mL^-1^ |
| 5-HT | Y=0.0085X | 0.9995 | 0.1 pg mL^-1^ |
| Glu | Y=0.0616X | 0.9996 | 0.1 μmol L^-1^ |
| DA | Y=0.0368X | 0.9993 | 0.1 pg mL^-1^ |
| LPS | Y=0.0028X | 0.9993 | 0.1 ng L^-1^ |

**Table S2 The sequence of primers for genes**

| Gene symbol | Forward | Reverse |
| --- | --- | --- |
| *β-actin* | 5'-CACCATGTACCCAGGCATTG-3' | 5'-CCTGCTTGCTGATCCACATC-3' |
| *Cd16* | 5'-CAGAATGCACACTCTGGAAGC-3' | 5'-GGGTCCCTTCGCACATCAG-3' |
| *Cd206* | 5'-CTCTGTTCAGCTATTGGACGC-3' | 5'-CGGAATTTCTGGGATTCAGCTTC-3' |
| *Cd68* | 5'-TGTCTGATCTTGCTAGGACCG-3' | 5'-GAGAGTAACGGCCTTTTTGTGA-3' |
| *Bdnf* | 5'-TCATACTTCGGTTGCATGAAGG-3' | 5'-AGACCTCTCGAACCTGCCC-3' |
